# Transcutaneous vagus nerve stimulation and extinction of prepared fear: A conceptual non-replication

**DOI:** 10.1038/s41598-018-29561-w

**Published:** 2018-07-31

**Authors:** Andreas M. Burger, Ilse Van Diest, Willem van der Does, Marsida Hysaj, Julian F. Thayer, Jos F. Brosschot, Bart Verkuil

**Affiliations:** 1 Institute of Psychology, Leiden University, Wassenaarseweg 52, 2333 AK Leiden, The Netherlands; 2 Faculty of Psychology, Katholieke Universiteit Leuven, Tiensestraat 102, 3000 Leuven, Belgium; 3 Department of Psychology, The Ohio State University, 1835 Neil Avenue Mall, Columbus, OH 43210 United States

## Abstract

Transcutaneous stimulation of the auricular branch of the vagus nerve (tVNS) may accelerate fear extinction in healthy humans. Here, we aimed to investigate this hypothesis in healthy young participants in a prepared learning paradigm, using spider pictures as conditioned stimuli. After a fear conditioning phase, participants were randomly allocated to receive tVNS (final *N* = 42) or sham stimulation (final *N* = 43) during an extinction phase. Conditioned fear was assessed using US expectancy ratings, skin conductance and fear potentiated startle responses. After successful fear acquisition, participants in both groups showed a reduction of fear over the course of the extinction phase. There were no between-group differences in extinction rates for physiological indices of fear. Contrary to previous findings, participants in the tVNS condition also did not show accelerated declarative extinction learning. Participants in the tVNS condition did have lower initial US expectancy ratings for the CS− trials than those who received sham stimulation, which may indicate an enhanced processing of safety cues due to tVNS. In conclusion, the expected accelerated extinction due to tVNS was not observed. The results from this study call for more research on the optimal tVNS stimulation intensity settings.

## Introduction

Increasing insights into the neurological underpinnings of fear have sparked an interest in neuromodulatory techniques aimed at enhancing fear extinction^1^. Notably, promising extinction-modulating effects have been found for various neurostimulation techniques that specifically target areas of the brain involved in extinction learning^2^. Among these techniques, stimulation of the vagus nerve (VNS) is of particular interest, as preliminary evidence from animal models and human fear conditioning studies point towards treatment-augmenting effects of VNS during exposure therapy^3–8^.

The first studies on the effects of vagus nerve stimulation on fear extinction were performed in rats. In two separate experiments, Peña and colleagues demonstrated that rats who received VNS displayed less freezing after extinction training than rats who had undergone sham surgery^3,4^. Decreased fear responses were also found during fear retention, two weeks after the initial extinction training^3^. These results were later replicated by the same research group, who showed that VNS improved the extinction of fear in rats by increasing the activation of the medial prefrontal cortex – basolateral amygdala pathway^8^.

VNS as a neuromodulatory add-on to extinction learning in humans has been an understudied subject up until now, because until recently VNS required surgical implantation of a neurostimulator. Recent studies have indicated that electrical stimulation of the concha of the left outer ear is a safe method to stimulate the auricular branch of the vagus nerve^9^. This transcutaneous VNS (tVNS) has similar effects on brain activation patterns as invasive VNS^10^ and increases performance in memory tasks and other cognitive tasks^11,12^. Although the working mechanisms of tVNS are currently still poorly understood^13^, invasive VNS is associated with the modulation of several neurotransmitters that could play an integral role in associative learning and memory. Firstly, VNS has been shown to increase levels of gamma-aminobutyric acid (GABA)^14^ and is associated with increased GABA receptor density^15^ in humans. GABA is the principal inhibitory neurotransmitter in the brain, and is associated with dampening fear learning. Although research on the effects of GABAergic activity on fear extinction is still somewhat limited, preliminary evidence suggests that increased GABAergic signaling would lead to decreased extinction learning and memory consolidation (for a review, see^16^). As such, the effects of invasive and transcutaneous VNS are unlikely to be mediated by GABAergic effects of the stimulation, as this would produce a general slowing in extinction rates, which is opposite of what has been found in previous studies^16^. Instead, a more likely working mechanisms for the effects of tVNS is through its afferent connection to the nucleus tractus solitaries (NTS), which activates the locus coeruleus to secrete norepinephrine (NE)^17–21^. NE is an important determinant of the extent to which salient (eg., threat and safety) memories are first encoded and subsequently consolidated in long term memory^22,23^. Importantly, the effects of NE on memory have been shown to be associated with activation of peripheral vagal afferents^24,25^ and thus provide a physiological basis for the potential effects of VNS on fear extinction in the present study.

The effects of tVNS on fear extinction in humans have been assessed in three previous studies. In the first study (*N* = 31)^6^, using a two-day protocol, participants who received tVNS showed accelerated declarative fear extinction learning compared to those who received sham stimulation on day one. No effects on retention of fear memories 24 h after extinction training were found. Effects of tVNS on physiological indices of fear could not be assessed due to technical issues and a lack of differential fear conditioning during the fear acquisition phase. A subsequent study (*N* = 39) used a three-day protocol with acquisition, extinction and retention of extinction on day 1, 2 and 3 respectively^7^. Participants who received tVNS again showed accelerated declarative fear extinction and no effects 24 h after fear extinction. In this study, no effects of tVNS on physiological indices of fear extinction were found, possibly indicating that tVNS affects fear extinction primarily via hippocampal, declarative pathways. Finally, another study tested the effects of tVNS on contextual fear conditioning in a virtual reality environment (*N* = 75, divided into a sham, tVNS, and no stimulation group)^26^. The study used a three day protocol. Contrary to the cue conditioning studies, no effects of tVNS were found on either declarative or physiological indices of fear, and no effects were found on fear retention. One possible caveat of these studies was the limited sample size, which reduced the statistical power to detect meaningful differences.

In the current study, we aimed to assess the effects of tVNS on both declarative and physiological fear extinction of cue-conditioned fear in a sample large enough to provide us with adequate statistical power to detect meaningful effects. Fear acquisition and extinction phases were conducted on the same day, similarly to one of our previous studies^6^.We conducted a randomized single-blinded controlled trial to compare the effects of tVNS and sham stimulation during the extinction of fear. Pictures of spiders were used as CS, as previous studies have indicated that these evolutionarily relevant stimuli lead to more pronounced fear responses and delayed fear extinction^27^. Similarly, other changes to the experimental paradigm were made, including the addition of a background noise and increased startle probe intensity (cf.^28^). High-intensity auditory stimuli are known to increase subjective and physiological arousal^29,30^, which in turn strengthens fear conditioning and subsequently slows down fear extinction^31^. These procedural changes were implemented to slow down fear extinction, thus allowing for a stronger differential effect of tVNS compared to sham stimulation. We hypothesized that tVNS would accelerate fear extinction, both on a declarative and a physiological level.

## Results

### Demographics

Out of the original ninety-seven participants, 1 participant was excluded because she wanted to stop the experiment pre-emptively out of fear for the spider pictures used as conditioned stimuli in the experiment. 4 participants had to be excluded due to mechanical failures with either the computer (*n = *1), the shock device (*n* = 2) or the tVNS device (*n* = 1). Finally, 7 participants were excluded because they had difficulty understanding the CS-US contingency during the Acquisition phase. Specifically, these participants either did not show higher average US-expectancy ratings for the final two CS+ trials than for the final two CS− trials (*n* = 4), or they reported US expectancy ratings below 50% for the CS+ trials during the final two trials (*n* = 3). The analyses described in this article were performed on the data of the remaining 85 participants (*N*_tVNS_ = 42 (out of which 5 were male), *N*_Sham_ = 43 (out of which 9 were male), *M*_age_ = 21.01 (*SD* = 1.87)).

As displayed in Table 1, there were no significant differences between experimental groups on background variables that may affect fear conditioning and extinction. Although participants in the tVNS group scored higher on the Abbreviated Spider Phobia Questionnaire (A-SPQ, difference not significant), participants in both groups still scored well within the range of a healthy sample^32^. Participants’ scores on trait worry (assessed using the Penn State Worry Questionnaire or PSWQ) and trait anxiety (assessed using the State-Trait Anxiety Questionnaire or STAI-Trait) were also comparable to norm scores from healthy college students or community samples^33^. State anxiety (assessed through the STAI-State) were slightly elevated compared to healthy college students or community samples (*M*_healthy norm_ = 35.2, *SD* = 8.4), but still well below state anxiety scores reported by clinical patient populations (*M*_psychiatry patients_ = 56.4, *SD = *13.8)^34^. Since these questionnaires were administered shortly after the acquisition phase, the elevated STAI-State scores in both groups may be a consequence of the fear conditioning procedure. Finally, no between-group differences were found on ratings of positive or negative mood.

**Table 1:** Descriptive statistics.

	tVNS *M*(*SD*)	Sham *M*(*SD*)	*p*
PSWQ	47.07 (8.59)	46.21 (9.98)	0.63
STAI state	41.83 (10.21)	41.28 (9.80)	0.95
STAI trait	38.44 (7.46)	37.51 (6.35)	0.48
A-SPQ	4.44 (3.43)	3.09 (2.78)	0.05
Positive mood	57.15 (15.41)	56.92 (19.35)	0.95
Negative mood	29.74 (18.25)	27.81 (18.23)	0.63
US unpleasantness Rating	65.14 (15.42)	63.54 (13.72)	0.61
Resting HRV (RMSSD)	45.61 (23.86)	43.91 (24.70)	0.75
Resting HR	75.10 (11.20)	76.64 (13.56)	0.57

*Note*. PSWQ: Penn State Worry Questionnaire, STAI-S: State-Trait Anxiety Questionnaire, A-SPQ: abbreviated Spider Phobia Questionnaire, US: Unconditioned stimulus, HRV: heart rate variability (Root mean square of the successive differences), HR: heart rate. Measurements of resting HR(V) were performed prior to the Acquisition phase. Between-group differences were tested using independent-samples t-tests.

No between-group differences were found on resting HR or HRV, which was assessed prior to the acquisition phase. Additionally, no differences were found after the Acquisition phase, when participants were asked to rate the unpleasantness of the US (see Table 1).

### Acquisition

Multilevel mixed model analyses were used to assess fear and extinction learning in our participants in terms of both self-reports and physiological outcomes. For a more detailed description of this statistical procedures, please refer to Statistical Analyses in the Methods section.

#### Expectancy Ratings

Participants showed clear signs of differential fear learning on US expectancy ratings during the acquisition phase, as reflected by the LogTrial*CStype interaction, *b* = 30.80, *t*(1269) = 11.74, *p* < 0.001 (see Table 2). Participants successfully learned that the CS− was safe, as reflected in the significant decrease in US expectancy ratings, *b* = 19.55, *t*(1269) = −10.05, *p* < 0.001. The significant main effect of CStype shows that US expectancies for the to-be-conditioned CS+ were already higher from the first trial, *b* = 25.06, *t*(1269) = 5.11, *p* < 0.001. This apparent ‘prior knowledge’ of the CS-US contingency can easily be explained by the standardized presentation order of CSs at the start of the acquisition phase: every acquisition phase started with a CS− trial, followed by a non-reinforced CS+ trial. Participants were instructed that one CS trial would never be followed by a shock, and therefore likely deduced that since the first trial was not followed by a shock, the second picture they saw would likely be the CS+.

**Table 2:** Regression weights and standard errors for mixed model analyses predicting US expectancy ratings in Acquisition and Extinction phases.

Predictor	Acquisition	Extinction
Intercept	40.70 (3.67)**	40.62 (4.18)**
CStype	25.06 (4.91)**	29.56 (5.51)**
LogTrial	−19.55 (1.94)**	−13.62 (1.64)**
LogTrial*CStype	30.80 (2.62)**	−11.61 (2.32)**
Condition	−2.32 (5.22)	−9.50 (5.95)*
Condition*CStype	0.70 (6.98)	10.08 (7.84)
Condition*LogTrial	2.42 (2.77)	3.16 (2.33)
Condition*LogTrial*CStype	−1.33 (3.73)	− 0.97 (3.30)

*Note*. Reference category for CStype is the CS− trial type. All analyses on the effects of tVNS were conducted using one-sided hypothesis tests. *p < 0.05, **p < 0.001.

As expected, we found no effects of Condition on US expectancy ratings during acquisition (all *p*s > 0.05).

#### Electromyography

Participants’ EMG responses reflected successful differential fear conditioning during the acquisition phase, as indicated by the significant differential decrease of CS− trials compared to CS+ trials, *b* = −0.58, *t*(1853) = −1.96, *p* = 0.05, as well as the differential decrease of ITIs compared to CS+ trials, *b* = −1.45, *t*(1853) = −4.87, *p* < 0.001 (see Table 3). There were no significant differences in EMG responses between the CS+ and the CS− or the CS+ and the ITI at the start of the acquisition phase (both *p*s > 0.05).

**Table 3:** Regression weights and standard errors for mixed model analyses predicting EMG in Acquisition and Extinction phases.

Predictor	Acquisition	Extinction
Intercept	57.03 (0.88)**	56.10 (0.80)**
CStype_CS−_	−1.76 (1.25)	1.12 (1.12)
CStype_ITI_	−1.51 (1.25)	−4.41 (1.12)
Trial	−0.16 (0.21)	−1.24 (0.12)**
Trial*CStype_CS−_	−1.45 (0.30)*	−0.09 (0.17)
Trial*CStype_ITI_	−0.58 (0.30)**	−0.07 (0.17)**
Condition	0.02 (1.26)	−0.47 (1.13)
Condition*CStype_CS−_	0.61 (1.78)	−1.32 (1.60)
Condition*CStype_ITI_	0.27 (1.78)	−0.02 (1.59)
Condition*Trial	−0.37 (0.30)	0.13 (0.17)
Condition*Trial*CStype_CS−_	0.64 (0.42)	0.03 (0.24)
Condition*Trial*CStype_ITI_	0.18 (0.42)	0.13 (0.24)

*Note*. Reference category for CStype is the CS+ trial type. All analyses on the effects of tVNS were conducted using one-sided hypothesis tests. *p < 0.05, **p < 0.001.

There were no significant between-group differences in EMG during the acquisition phase (all *p*s > 0.05).

#### Skin Conductance Responses

Participant’s SCR reflected a clear differential learning curve, where SCR habituated over time for both CS+ and CS− trials as reflected by the main effect of LogTime, *b* = −0.17, *t*(1224) = −10.21, *p* < 0.001, but CS+ trials showed a differential increase compared to CS− trials over the course of the acquisition phase, *b* = 0.08, *t*(1224)* = *4.87, *p* < 0.001 (see Table 4). Initial responses to the CS+ were lower than to the CS−, as reflected by the main effect of CStype, *b* = −0.08, *t*(1224) = −2.89, *p* = 0.01. The initial difference between CStypes likely reflects the non-randomized initial order of CS presentations: the first trial of the acquisition phase was always a CS− trial.

**Table 4:** Regression weights and standard errors for mixed model analyses predicting SCR in Acquisition and Extinction phases.

Predictor	Acquisition	Extinction
Intercept	0.41 (0.04)**	0.13 (0.02)**
CStype	−0.08 (0.05)**	0.01 (0.02)
Trial^a^	−0.17 (0.02)**	−0.01 (0.002)**
Trial^a^*CStype	0.08 (0.02)**	−0.001 (0.002)
Condition	−0.05 (0.05)	−0.02 (0.03)
Condition*CStype	−0.01 (0.03)	0.01 (0.02)
Condition* Trial^a^	0.02 (0.02)	0.004 (0.003)
Condition* Trial^a^ *CStype	−0.002 (0.02)	−0.002 (0.003)

*Note*. Reference category for CStype is the CS− trial type. All analyses on the effects of tVNS were conducted using one-sided hypothesis tests. *p < 0.05, **p < 0.001.

^a^Trial variable was log transformed in the Acquisition model.

There were no significant between-group differences in SCR during the acquisition phase (all *ps* > 0.05).

### Extinction

#### Expectancy Ratings

Participants in both groups showed a clear differential declarative fear response at the start of the extinction phase, as reflected in the significant main effect of CStype, *b* = 29.56, *t*(1949) = 5.36, *p* < 0.001 (see Table 2). US expectancy ratings for both CS types decreased over the course of the extinction phase, *b* = −13.62, *t*(1949) = −8.30, *p* < 0.001. Expectancy ratings for CS+ trials showed a stronger decline than CS− trials, *b* = −11.61, *t*(1949) = −5.00, *p* < 0.001, indicating extinction learning.

**Figure 1:**
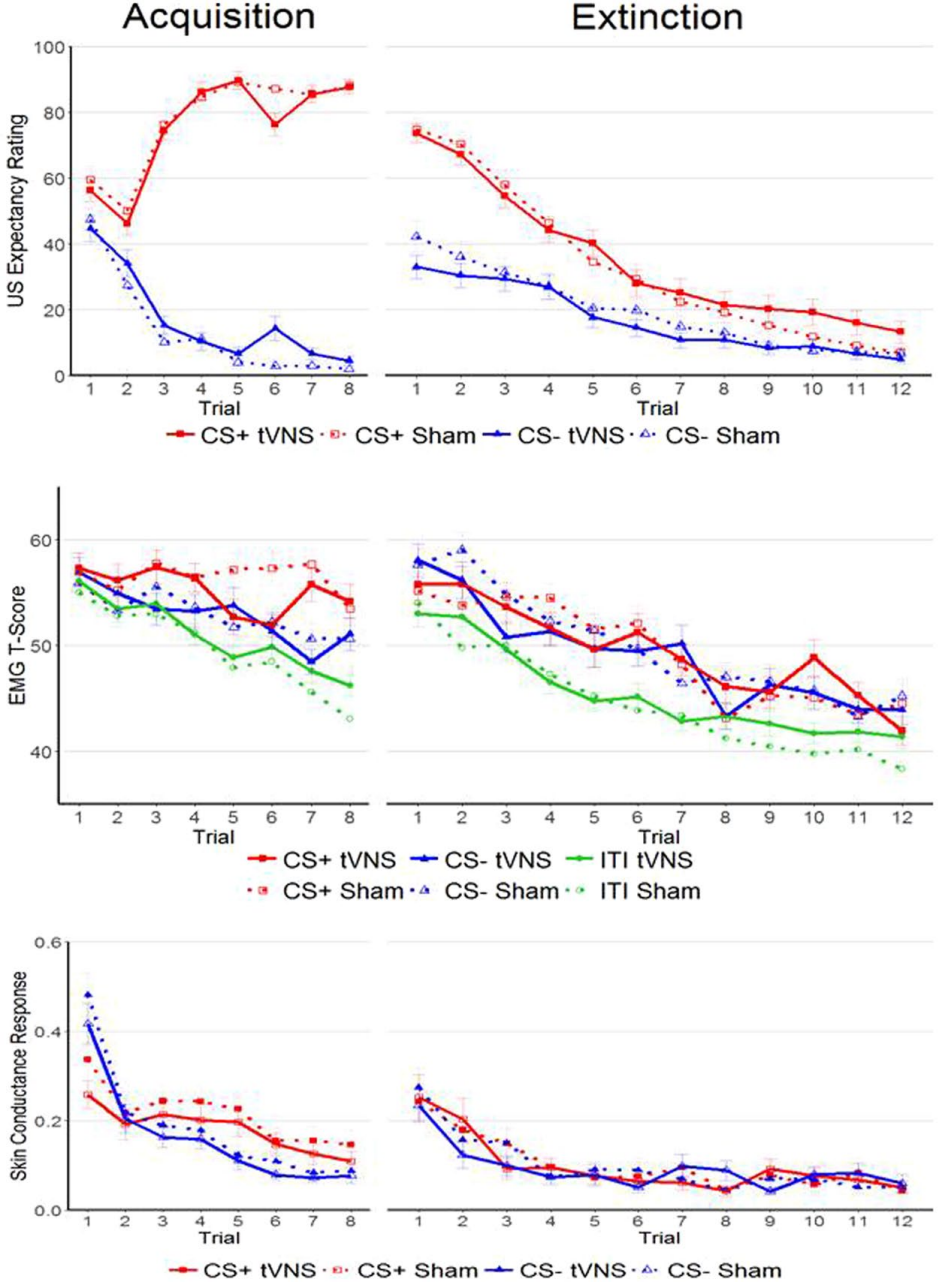
Overview of results for the acquisition (left) and extinction (right) phases of the study. The figure shows mean responses per trial for US expectancy ratings (top), EMG (middle) and SCR (bottom). Error bars indicate ±1 standard error of the mean.

There were no significant effects of Condition on learning curves for CS+ trials or CS− trials (both *p* > 0.05). However, there was a significant main effect of Condition on US expectancy ratings, *b* = −9.51, *t*(83) = −1.60, *p* = 0.05, δ = 0.36, reflecting lower US expectancy ratings in the tVNS condition. This main effect of Condition should be interpreted with caution, as the regression weights of the non-significant interactions of Condition*LogTrial (*b* = 3.16, *t*(1949) = 1.36, *p* = 0.91) and Condition*CStype (*b* = 10.09, *t*(1949) = 1.29, *p* = 0.90) indicate that the significant effect of Condition specifically reflects lower US expectancy ratings in the tVNS condition for CS− trials at the start of the extinction phase (see Fig. 1).

#### Electromyography

Participants showed strong overall startle responses at the start of the extinction phase, irrespective of CStype, as indicated by the overall intercept, *b* = 56.10, *t*(2799) = 70.52, *p* < 0.001 (see Table 3). They displayed a significant differential fear response to the CS+ compared to the ITI, *b* = 4.41, *t*(2799) = 3.92, *p* < 0.001. However, they did not show differential responding when comparing the CS+ trial to the CS− trials, *b* = 1.12, *t*(2799) = 0.99, *p* = 0.32, possibly indicating a generalization of the fear memory. In subsequent trials, we see a significant decrease in startle responses as indicated by the main effect of Trial, *b* = −1.24, *t*(2799) = −10.38, *p* < 0.001. However, there was no significant differential learning curve for CS+ trials either in comparison to ITIs or CS− trials (both *p* > 0.05). Thus, although participants displayed a strong general decrease in fear potentiated startle responses, participants did not show differential extinction learning.

There were no significant effects of Condition on initial EMG or on EMG learning curves over the course of the extinction phase (all *p* > 0.05, see Table 3 for regression weights).

#### Skin Conductance Responses

Participants did not show significant differential fear responses at the start of the extinction phase (main effect CStype, *p* = 0.67, see Table 4). Specifically, as displayed in Fig. 1, participants in both conditions had larger SCR at the start of the extinction phase compared to the end of the acquisition phase, irrespective of CStype. Over the course of the extinction phase, SCR decreased significantly, *b* = −0.01, *t*(1875) = −4.00, *p* < 0.001, irrespective of CS type (interaction CStype*Trial, *p* = 0.64). Although this non-differential reduction in SCR may reflect the extinction of fear, it is difficult to disentangle this effect from a more general habituation response that was also evident during the acquisition phase. There were no significant effects of Condition on initial SCR or on SCR learning curves over the course of the extinction phase (all *p* > 0.05, see Table 4 for regression weights).

### Side-effects

Using a short form of seven potential side-effects that we have observed in prior studies, we asked participants to rate their sensations of the neurostimulation at the end of the extinction phase while the stimulation was still active. Although participants in the tVNS condition reported higher side-effect intensity levels on average, it should be noted that average side-effect ratings were relatively low in both groups (overall *M*_tVNS_ = 2.20(0.65), *M*_Sham_ = 1.90(0.71), *t*(87) = −2.01, *p* = 0.05).

### Exploratory Analyses

We conducted additional exploratory analyses to assess possible moderators of the effects of tVNS on US expectancy ratings during the extinction phase. Specifically, the questionnaires that participants had completed in between the acquisition and extinction phases (PSWQ, STAI-S, STAI-T and SPQ), as well as baseline RMSSD, were added to the model described in section 3.2 to see whether they moderate the effects of tVNS on declarative fear extinction. These factors were selected as potential moderators because their underlying constructs (i.e. perseverative cognition, state and trait anxiety, and vagal tone) have been associated with fear and extinction learning in previous studies (e.g.^35–38^). All factors were added separately to the model, both as continuous and as median-split variables. However, none of these variables improved the overall model fit or resulted in significant interactions between the moderator and Condition. None of the possible moderators provided main effects for US expectancy ratings, either. Thus, we can conclude that in our current sample, RMSSD nor anxiety at baseline significantly affected the effects of tVNS.

## Discussion

We tested the effects of tVNS on fear extinction learning in humans in a single-day fear conditioning procedure. Based on previous research^6,7^, we expected accelerated fear extinction after tVNS. The results showed no effect of tVNS on the rate of declarative fear extinction nor on any of the physiological indices of fear. We did find a small effect of tVNS on US expectancy ratings for CS− trials at the start of the extinction phase.

The lack of effects of tVNS on declarative fear extinction learning was unexpected, as this seems to contradict findings from our previous studies^6,7^. There they are in line, however, with a recent study which found no effects of tVNS on contextual fear extinction in a virtual reality environment^26^. The current study was designed to be a more highly powered conceptual replication of our previous studies. However, there were several differences between the paradigm of the current study and the ones used in the previous studies. First, in the current study, we used pictures of spiders instead of geometrical shapes as CSs. Previous studies have indicated that spiders and other evolutionarily relevant threat pictures may lead to stronger acquisition of fear and slower extinction learning^32^. Other changes we made to the paradigm included adding a 70 dB background noise and increasing the intensity of the startle probe (104 dB instead of 100 dB and 95 dB). All changes were made to promote a high arousal level in participants, which would lead to a stronger acquisition of fear, and – theoretically - allowing tVNS to make a larger difference.

In line with the expected increased arousal experienced by participants in this study, participants in the sham condition reported higher US expectancy ratings for CS− trials compared to previous studies. Additionally, participants in both groups showed a strong, non-differential increase in SCR and startle responding at the start of the extinction phase compared to the end of the acquisition phase. These increased non-differential fear responses at the start of the extinction phase clearly reflect the increased apprehensiveness of participants in this study, and may partly explain the discrepancy in the results from this study compared to our previous studies. Considering that the expected working mechanism of tVNS is through the modulation of noradrenergic activity, one possible explanation for the lack of effects of tVNS on fear extinction learning is that the vagus nerve had been activated through adrenergic pathways in both conditions, as a result of the increased arousal experienced by participants in our current conditioning paradigm. Indeed, administration of peripheral adrenaline leads to an increased firing rate of vagus nerve fibers in rats^39^. In turn, administration of peripheral adrenaline prior to the extinction phase has been associated with stronger extinction learning in mice, possibly due to subsequently increased central noradrenergic activity^40^. Clearly, there is a need for more fundamental studies on the working mechanisms of tVNS in humans and its interactions with background levels of arousal, since this could strongly affect the clinical applicability of tVNS.

The lower initial US expectancy rating for CS− trials in the tVNS condition was an unexpected finding, since our previous studies found effects of tVNS on the learning rates of the CS+, not the CS−. This effect of tVNS on CS− ratings at the start of the extinction may simply reflect baseline differences between participants, independent of the experimental manipulation. Alternatively, this effect may reflect an improved ability of participants in the tVNS condition to immediately recognize the CS− as a safety cue. This result would be in line with the Generalized Unsafety Theory of Stress (or GUTS^41,42^), which posits that vagal activity is an important determinant of the maintenance of prefrontal inhibition of the stress response once safety is detected. As such, vagus nerve activation may increase a person’s ability to identify and remember that a situation is indeed safe and can prevent a stress response from generalizing from a certain stimulus (e.g. the CS+) to a wider context (e.g. the CS−). Indeed, Fig. 1 shows a clear increase in US expectancy ratings for CS− trials at the start of the extinction phase compared to the end of the acquisition phase, indicating a generalization of the fear response and an increase in the uncertainty about CS−US contingencies. Even though we did not formally hypothesize this effect to occur based on previous findings, the results found in this study are clearly in line with the GUTS and could point towards an interesting therapeutic effect of tVNS. Further research is clearly warranted to test whether these results can be corroborated.

One could argue that groups may not have been similar on their abbreviated SPQ score, and participants in the tVNS condition reported slightly higher symptoms of spider phobia than the sham condition. However, it’s important to note that participants in both conditions scored well within the normal range and should not be classified as spider phobics. As such, we do not believe that differences in spider phobia are likely to explain the lack of effects of tVNS found in this study.

The current study included mainly female participants, which may have possibly limited the generalizability of the findings. Although research on this topic is limited, previous studies in animals^43^ and in humans^44,45^ have found no consistent differences on vagus nerve morphology between males and females. However, effects of tVNS on LC-NE activity may be different for men and women due to differences in morphology of the LC and CRF1 receptors^46^. Notably, LC dendrites in female compared to male rats are longer and more complex^47^, which could lead to a stronger information relay from the NTS (the main terminal of vagal afferents) to the LC^48^. While these intricate differences in LC dendrite morphology have not yet been studied in humans, possible sex differences in the sensitivity of the LC to changes in afferent signaling of the vagus to the NTS clearly warrant additional research. With respect to our current study, we cannot be certain whether the skewed male-to-female participant ratio has affected the results of our analyses. One important argument that we’ve made before^6^, is that not much is known about the optimal stimulation intensity for human auricular tVNS. The stimulation intensity used in this study (0.5 mA) is based on invasive VNS studies that found cognitive effects using this stimulation intensity. An important difference between invasive and transcutaneous VNS is that during invasive VNS, the stimulation coil is wrapped directly around the vagus nerve. During tVNS, the stimulation current first has to pass a layer of skin tissue before diffusely reaching the vagus nerve. Thus, the electrical current is impeded by skin, leading to a smaller overall electrical current reaching the vagus nerve and a larger between-participant variability in the amount of electrical current that does reach the nerve, based on inter-individual differences in impedance. These factors may have reduced the effects tVNS may have had on extinction learning. This further highlights the need for more fundamental studies of optimal stimulation intensities but also of biomarkers of afferent vagus nerve activation.

In summary, in this study we found no indications that tVNS accelerated the extinction of conditioned fear. However, participants who received tVNS displayed lower US expectancy ratings to the CS− trials at the start of the extinction phase compared to participants in the sham condition. This effect was not expected beforehand and may reflect a coincidental finding. On the other hand, it is in line with the GUTS model of anxiety, which posits that the vagus nerve plays an integral part in recognizing safety signals in the environment. The results from this study clearly call for more elaborate studies which focus on the ideal tVNS stimulation settings, the comparability of transcutaneous and invasive VNS, and search for possible biomarkers to non-invasively assess vagus nerve activity in humans.

## Methods

### Participants

We conducted a sample size calculation beforehand to estimate the number of participants required to detect a medium effect size for the main effect of condition in a multilevel analysis. This calculation indicated that given a power of 1 − β = 0.80, a significance level of α = 0.05, 12 repeated measurements during the extinction phase and a minimum effect size of δ = 0.5, we needed at least 35 participants in each condition^49^.

Eligible participants were healthy college students between the ages of 18 and 25. Participants with spider phobia, epilepsy, bradycardia, cardiac arrhythmia, cardiac diseases, significant head trauma, pregnancy, drug use, neurological or psychiatric disorders were excluded from participating in this study. Participants received either course credits or 10 euro as compensation for participating in the study. The study was approved by the Institutional Ethical Board of Leiden University, Institute of Psychology (CEP #4782302709). The experiment was performed in accordance with relevant guidelines and regulations. All participants gave their written informed consent prior to the start of the experiment.

### Stimuli and Apparatus

#### Stimuli

CSs were pictures of spiders (IAPS numbers 1200–1201, based on^50^). The slides were 18 cm high and 25 cm wide and were presented on a 17-inch CRT monitor in the middle of the screen on a black background. Both CSs were presented for 8 seconds. During the acquisition phase, one of the CSs was followed by the US in 75% of the trials (CS+), while the other CS was never followed by a US (CS−). To-be conditioned stimuli were assigned as CS+ and CS− in a counterbalanced order. The US occurred 7.5 s after CS+ onset. Intertrial interval durations varied randomly between 15 and 25 seconds. Presentation of CSs was semi-randomized, to ensure that one CS type could not be presented on more than three subsequent trials.

The US was a 20 ms electric shock that was delivered to the wrist of the non-dominant hand. A conductive gel was used between the electrodes and the skin. The shock was delivered using a Grass S48 stimulator. Shock intensity was determined at the start of the experimental procedure. The intensity was individually set at a level that was very uncomfortable, but not painful. Participants received shocks of gradually increasing intensity, starting at 1 mA and increasing in 5 mA increments. After every shock, participants were asked to rate what they had felt and whether the shock intensity would have to be increased to reach a level that was ‘very uncomfortable, but not painful’. Once participants felt that they had reached a shock intensity that corresponded to this level, the shock intensity was kept stable at this level for the rest of the experiment.

The startle probe consisted of a 50 ms, 104 dB burst of white noise with near instantaneous rise time that was administered to both ears via headphones. Startle probes were presented 7 seconds after every CS and intertrial interval (ITI) onset. Throughout the acquisition and extinction phases, participants also heard a continuous background noise of 70 dB pink noise from their headphones. Both the startle probes and the continuous background noise were created using Audacity 2.0.2 software.

#### tVNS and sham stimulation

Transcutaneous vagus nerve stimulation (tVNS) is a non-invasive method of electrically stimulating the afferent auricular branch of the vagus nerve located at the cymba conchae^9^.

We used a tVNS device that provides electrical stimulation using two titanium electrodes, positioned on top of a silicon earplug, which are connected by a wire to a portable neurostimulator (Nemos®, Cerbomed, Erlangen, Germany). The electrodes deliver 30-second waves of electrical stimulation (0.5 mA, 25 Hz, 250 μs wavelength) to the concha of the left outer ear^51^, alternated by 30-second breaks. In the sham condition, the electrodes are connected to the center of the earlobe instead of the concha^51^. The stimulation parameters (current, frequency, on/off cycle) were fixed for all participants. We stimulated the left ear to avoid potential cardiac effects that have been related to efferent vagal fibers of the right ear^52^ but not the left^9^.

#### Expectancy Ratings

Participants were asked to rate the extent to which they expected a shock to occur during every CS presentation using a visual analogue scale that ranged from 0 (‘not at all’) to 100 (‘certainly’). Participants were instructed to give these ratings by moving the cursor within 5 seconds after CS onset, after which the scale would disappear from the screen. The scale was presented at the bottom of the screen so as not to draw too much attention away from the stimuli. At the beginning of every new CS presentation, the slide would reappear and the cursor would return to the ‘uncertain’ middle position (cf.^53^).

#### Psychophysiological Measures

We measured the potentiation of the eyeblink startle reflex to an acoustic startle probe by using electromyography (EMG) of the left orbicularis oculi muscle. To measure the eyeblink reflex, we used two 4 mm Ag-AgCl Biopac electrodes, one placed below the lower left eyelid in line with the pupil in forward gaze, and the second one placed approximately 1 cm lateral to the first (in accordance with the guidelines specified in^54^). EMG was measured using a Biopac system, and filtered by 500 Hz low-pass and 10 Hz high-pass hardware filters. The EMG signal was grounded by the electrodermal electrodes. The raw response signals were visually checked by the first author in a blinded procedure, and trials that were affected by movement artifacts or overall poor signal quality were manually removed (0.5% of trials).

EMG responses were calculated by subtracting the mean EMG signal in the 20 ms period directly following the startle probe presentation from the maximum EMG amplitude within the response window between 21–150 ms following startle probe onset^54^.

Electrodermal activity was measured using two Ag/AgCl electrodes (Biopac EL507-10). The electrodes were attached to the distal phalanges of the index and middle finger of the nondominant hand^55^.

The skin conductance response (SCR) in response to the CS was determined by subtracting the average baseline skin conductance level (2 s before CS onset) from the peak skin conductance level in the first 6 seconds following CS onset. Responses lower than 0.02 micro Siemens were scored as zero and remained in the analyses^56^. SCRs were further log transformed to normalize the data distribution.

#### Cardiac activity

Heart rate (HR) and heart rate variability (HRV) were derived from the raw ECG signal, which was measured continuously using a two-lead set-up of the Biopac system. The ECG signal was grounded by the electrodermal electrodes. The raw ECG signal was measured at 1000 Hz and subsequently filtered using 2 Hz low-pass and 50 Hz high-pass software filters. The signal was subsequently visually inspected checked by the first author in a blinded procedure and artifacts were manually corrected. Interbeat intervals were extracted from the filtered signal, from which HR and the root mean square of the successive differences (RMSSD) between heart rates were calculated using a custom Matlab script. A five-minute baseline recording of every participant’s RMSSD level was used to assess participants’ vagally-mediated HRV and to check for possible differences in baseline vagal tone.

#### Questionnaires

The State Trait Anxiety Inventory (STAI) is a self-report questionnaire consisting of 2 scales with 20 questions each, measuring both state and trait anxiety^34,57^. The STAI has shown high internal consistency and validity^57,58^. The range of both scales of the STAI is between 20 and 80. Norm scores from the general population are 33.16 for the state scale and 36.35 for the trait scale.

The Penn State Worry Questionnaire (PSWQ) is a 16-item self-report questionnaire that assesses the duration and uncontrollability of worry^59^. The PSWQ has demonstrated high reliability, temporal stability and validity in the assessment of trait-worry^59,60^. The range of the PSWQ is between 16 and 80. A PSWQ score of 62 has been validated as a screening tool for generalized anxiety disorders^61^.

The Abbreviated Spider Phobia Questionnaire (SPQ) is a self-report questionnaire consisting of 15 yes-or-no questions that assess the subjects fear of spiders^62^. Since pictures of spiders were used as conditioned stimuli, between groups differences in spider phobia severity may affect fear and extinction learning rates. The abbreviated SPQ has shown high internal consistency and strong discriminatory validity^32^. Scores on the abbreviated SPQ range between 0 and 15, with spider phobic participants scoring significantly higher than nonphobics (*M*_phobics_ = 10.31, *M*_non-phobics_ = 2.06)^32^.

Participants rated their current mood (happiness, anxiety, irritableness, sadness) on a visual analogue scale ranging from (0) ‘not at all’ to (100) ‘completely’. The scores on these scales were converted into two comprehensive scores, ‘positive affect’ (score on the happiness subscale) and ‘negative affect’ (mean score on anxiety, irritableness and sadness subscales). Visual analogue scales are brief and valid measurements of mood state^63^.

At the end of the experiment, participants rated potential negative side-effects as a result of the stimulation on a scale of 1 (“applies not at all”) to 5 (“completely applies to me”) (cf.^6,7^). Side-effects included in the list were headache, pain in the neck, nausea, muscle contractions in the face or neck, prickling sensation under the electrodes, burning sensation under the electrodes and a general feeling of discomfort. Both the number of side effects (scores above 1 were counted as a side effect) and the mean intensity of the side effects were compared between the groups.

### Experimental Procedure

At the start of the experimental procedure, the electrodes for EMG, SCR and ECG recordings was attached to the participant’s skin. The shock device was then attached to the participant’s non-dominant wrist, after which the shock intensity was individually determined.

Participants were told that they would see two pictures, and it was their task to learn to predict which one was often followed by a shock and which one was not. As such, this design included a partial instruction on CS-US contingencies, which leads to a more uniform fear learning compared to a uninstructed fear study, while still leaving enough room for associative learning to take place^64^. Prior to the start of the acquisition phase, a five-minute baseline measurement of every participant’s RMSSD level was recorded to assess participants’ vagally-mediated HRV, during which time participants watched a muted neutral film clip.

We included a habituation phase prior to the acquisition phase to ensure that participants habituated to the stimuli used in the paradigm prior to differential associative fear learning^64^. Participants were informed that in this phase, they would be introduced to the different stimuli that would be presented in the rest of the task. First, we presented both CS pictures once. Subsequently, we presented 10 startle probes over a period of 150 seconds to habituate startle blink responses. During this period, participants were also habituated to the background noise, which would stay on for the remainder of the Acquisition and Extinction sessions, although it was temporarily switched off while participants filled in questionnaires and while the tVNS device was attached.

During the acquisition phase, both the CS+ and the CS− were presented eight times. The acquisition phase for every participant started with a CS− trial, followed by a CS+ trial. The CS+ was followed by the US in 75% of the trials – specifically, the first and the fifth presentation were always unreinforced (cf.^50,65^). The CS− was never followed by a shock.

At the end of the acquisition phase, participants were asked to rate the unpleasantness of the US on a scale from 0 (not unpleasant at all) to 100 (very unpleasant).

After the acquisition phase, we attached the tVNS device to the ear of the participant and we started either tVNS or sham stimulation. Participants were sequentially assigned to receive either tVNS or sham stimulation to reduce the odds of unbalanced group sizes. Regardless of experimental allocation, participants were told that stimulation was expected to affect physiological processes during the tasks. Participants wore the nerve stimulator throughout the rest of the experimental procedure. Prior fMRI studies have noted a temporal latency in the neurological effects of tVNS^10^, which is why we instructed participants to complete a short demographics questionnaire and several other questionnaires with the tVNS device in place and active, before starting the extinction phase. Completing the questionnaires took roughly 12 minutes.

**Figure 2:**
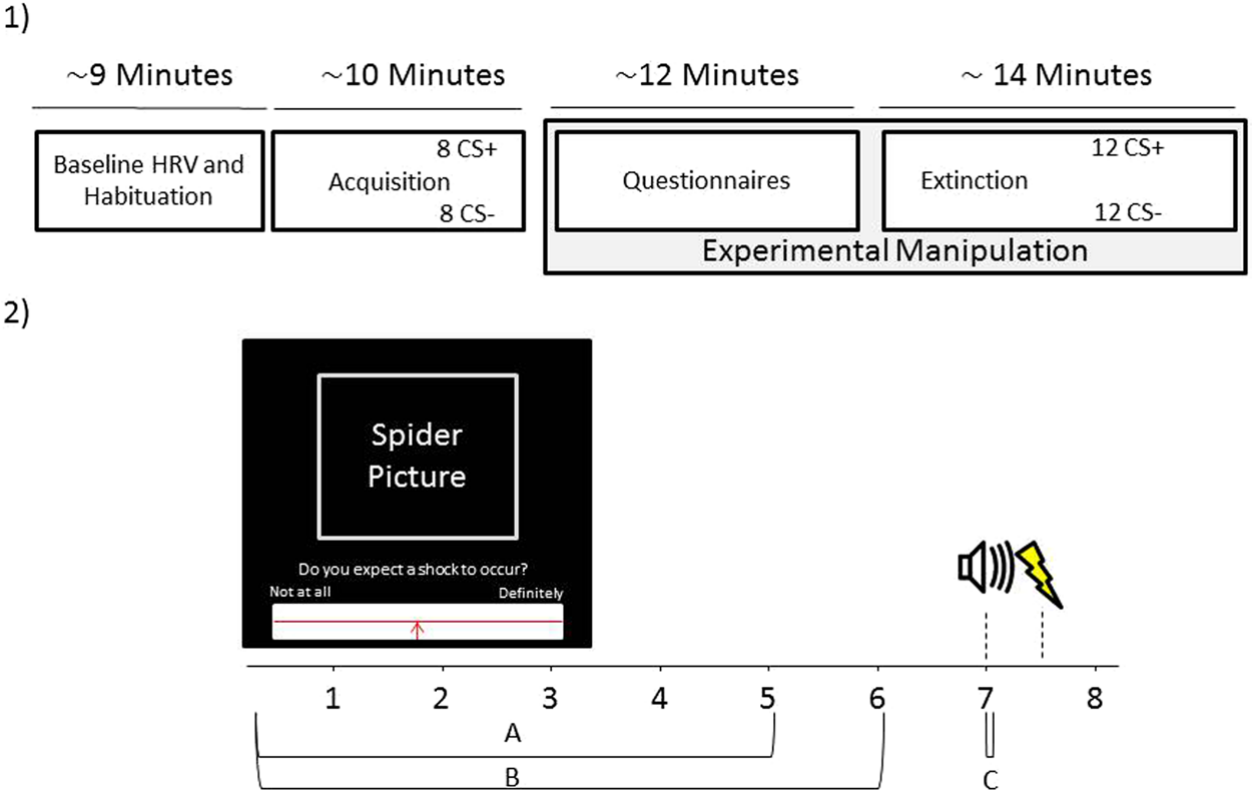
Experimental Overview. (**1**) The overall experiment lasted approximately 45 minutes and could be broadly subdivided into a baseline phase, an acquisition phase, a phase where participants filled in some questionnaires and finally an extinction phase. Participants received tVNS or sham stimulation only in the last two phases. (**2**) Every trial lasted 8 seconds in total. Participants were asked to rate to what extent they expected a shock to occur within the first 5 seconds of CS onset (response window A). Maximum skin conductance responses were recorded within the first 6 seconds (response window B) and maximum startle responses were recorded within 21–150 ms after startle probe onset (response window C).

The extinction phase consisted of 12 presentations of both CS+ and CS− trials. Both CS types were unreinforced during the extinction phase. At the end of the extinction phase, participants reported any potential side-effects from the nerve stimulation procedure. Afterwards, the tVNS device was removed from the participant’s ear. On average, the experimental session lasted roughly 50 minutes, during which participants received electrical stimulation to their ear for roughly 25 minutes. See Fig. 2 for an overview of the experimental procedure.

### Statistical Analyses

Between-group differences on all baseline questionnaires and baseline HRV data were analyzed using independent samples *t*-tests.

Multilevel mixed model analyses were used to assess whether the conditioning procedure resulted in successful fear learning in our participants in terms of both self-reports and physiological outcomes. After we ascertained that participants showed a significant response differentiation between CS− and CS+ trials on an index of fear during acquisition, we continued to use multilevel mixed model analyses to analyze the effects of tVNS during the extinction phase.

All multilevel mixed models were created using maximum likelihood modeling. We allowed intercepts to vary randomly across participants. Adding random slopes did not improve model fit and were thus removed from all models. We modeled the error covariance structure of the repeated measurements (every trial was nested within CStypes, which were in turn nested within individual participants) by specifying a heterogeneous AR1 autoregressive structure.

The independent variable Trial, signifying trial number within each session, was group mean centered around the first trial of every phase. CStype was dummy-coded, using CS− trials as the reference category for SCR and US expectancy ratings and using CS+ trials as the reference category for EMG, to allow comparisons of CS+ trials with both CS− trials and ITI.

To account for possible non-linear learning rates, we fitted linear and loglinear time curves to all models, as we did previously^6,7^, and removed either of these variables if this resulted in better model fit according to BIC estimates.

Cohen’s d effect sizes were calculated for significant effects of tVNS using the formula $d=\frac{{\rm{b}}}{{\rm{pooled}}\,S{\rm{D}}^{\prime} }$ where b denotes the regression coefficient of the corresponding effect and SD corresponds to the pooled within-group standard deviation^66^.

All analyses concerning the effects of tVNS on extinction learning are reported as one-tailed tests to increase our power to detect an effect in the direction we expected. Analyses were conducted using the *nlme* and *lmerTest* packages in *R*.

Additionally, we performed post-hoc Bayesian re-analyses of the effects of tVNS during the extinction phase. The results of these re-analyses support the main analyses and are presented in a Supplementary file.

The datasets analyzed during the current study are available on the Open Science Framework, osf.io/p2wfc.

## Electronic supplementary material

Supplementary File - Bayesian reanalysis
