# Supplementary material for: Transcutaneous vagus nerve stimulation and extinction of prepared fear: A conceptual non-replication
Source: Sci Rep. 2018 Jul 31;8:11471. doi: 10.1038/s41598-018-29561-w (PMC6068181; doi:10.1038/s41598-018-29561-w)
Supplement: Supplementary file 1 — Supplementary File - Bayesian reanalysis [file 41598_2018_29561_MOESM1_ESM.docx]

Transcutaneous vagus nerve stimulation and extinction of prepared fear:

A conceptual non-replication

Supplementary File

Andreas M. Burger^a,b^*, Ilse Van Diest^b^**,** Willem van der Does^a^, Marsida Hysaj^a^, Julian F. Thayer^c^, Jos F. Brosschot ^a^, Bart Verkuil^a^

^a^ Institute of Psychology, Leiden University; [a.m.burger@fsw.leidenuniv.nl](mailto:a.m.burger@fsw.leidenuniv.nl), [vanderdoes@fsw.leidenuniv.nl](mailto:vanderdoes@fsw.leidenuniv.nl), [m.hysaj@fsw.leidenuniv.nl](mailto:m.hysaj@fsw.leidenuniv.nl), [brosschot@fsw.leidenuniv.nl](mailto:brosschot@fsw.leidenuniv.nl), [bverkuil@fsw.leidenuniv.nl](mailto:bverkuil@fsw.leidenuniv.nl). Wassenaarseweg 52, 2333 AK Leiden, The Netherlands.

^b^ Faculty of Psychology, Katholieke Universiteit Leuven; [ilse.vandiest@kuleuven.be](mailto:ilse.vandiest@ppw.kuleuven.be). Tiensestraat 102, 3000 Leuven, Belgium.

^c^ Department of Psychology, The Ohio State University; [thayer.39@osu.edu](mailto:thayer.39@osu.edu). 1835 Neil Avenue Mall, Columbus OH 43210, United States.

*Andreas M. Burger is the Corresponding Author for this manuscript.

Bayesian re-analysis

In the main manuscript, we tested the effects of tVNS on fear extinction within a null-hypothesis significance testing (NHST) framework. In short, the NHST framework tests the likelihood that one would gather certain data under the assumption that the null hypothesis is true. Significant results allow researchers to reject the null hypothesis, but non-significant results do not allow us to accept the null, as the validity of the null hypothesis is an underlying assumption of the test and not something that is directly tested. Alternatively, Bayesian analyses allow researchers to test the likelihood of either the null hypothesis or the alternative hypothesis, *given the data*. Here, we will re-analyze the effects of tVNS on the extinction of fear using a repeated measures Bayesian analysis in *JASP* (version 0.8.6)^1,2^.

In the following sections, we will report the results of the Bayesian re-analyses, focusing on the effects of tVNS on declarative and physiological fear extinction. We will focus on reporting the Bayes Factors, which reflect a ratio of the likelihood that the data fit under the null hypothesis compared to the likelihood that the data fit under the alternative hypothesis. Specifically, we will report the BF_01­_, with higher values reflecting more evidence in support of the null hypothesis.

As these analyses were conducted post-hoc, no changes were made to the default priors given by JASP (*r* scale fixed effects = 0.5, *r* scale random effects = 1, *r* scale covariates = 0.354). These priors constitute a non-informative uniform prior distribution^3^.

In all instances, the null model consisted of a model that included the terms CStype, Trial, and Trial*CStype, as well as a random intercept for every subject. This null model was compared with models that included the Condition*CStype*Trial interaction and all lower order interactions and main effects.

**Results**

Expectancy Ratings

The results of this study strongly support the hypothesis that tVNS did not affect the extinction of declarative fear. When comparing the null model to the ‘full’ alternative model including Condition and its interactions with CStype and Trial, the Bayes Factor indicated that these data were over 100,000 times more likely to be observed under the null hypothesis (BF_01_ = 122,654, see table 1). Simpler models that did not include higher order interaction terms between Condition and CStype and/or Trial resulted in smaller BF_01_, but the data supported no model that contained the Condition term compared to the null model.

Fear Potentiated Startle Responses

The results of this study strongly support the hypothesis that tVNS did not affect the extinction of fear potentiated startle responses. When comparing the null model to the ‘full’ alternative model including Condition and its interactions with CStype and Trial, the Bayes Factor (BF_01_ = 4.823e^6^, see table 1) indicated that these data were over 4,000,000 times more likely to be observed under the null hypothesis. Simpler models that did not include higher order interaction terms between Condition and CStype and/or Trial resulted in smaller BF_01_, but the data supported no model that contained the Condition term compared to the null model.

Skin Conductance Responses

The results of this study strongly support the hypothesis that tVNS did not affect the extinction of skin conductance responses. When comparing the null model to the ‘full’ alternative model including Condition and its interactions with CStype and Trial, the Bayes Factor (BF_01_ = 1.624e^6^, see table 1) indicated that these data were over 1,000,000 times more likely to be observed under the null hypothesis. Simpler models that did not include higher order interaction terms between Condition and CStype and/or Trial resulted in smaller BF_01_, but the data supported no model that contained the Condition term compared to the null model.

**Discussion**

The Bayesian re-analyses provide very strong support for the null model, which corroborates and extends the results from the main analyses. The null model, which posits that tVNS did not affect individuals’ ability to learn in this trial, was supported by strong evidence for both physiological and declarative indices of fear.

**References**

1. Morey, R. D. & Rouder, J. N. BayesFactor (Version 0.9.10-2). (2015).

2. JASP Team. JASP (Version 0.8.6). (2018).

3. Rouder, J. N., Morey, R. D., Speckman, P. L. & Province, J. M. Default Bayes factors for ANOVA designs. *J. Math. Psychol.* **56,** 356–374 (2012).

**Table 1.** Model comparisons of Bayesian RM ANOVAs of US expectancy ratings with and without Experimental Condition.

| **Models** | **P(M)** | **P(M\|data)** | **BF _M_** | **BF _01_** | **Error %** |  |
| --- | --- | --- | --- | --- | --- | --- |
| Expectancy Ratings |  |  |  |  |  |  |
| Null model (incl. CS Type, Trial, CS Type * Trial, Subject) | 0.167 | 0.52 | 5.51 | 1.00 |  |  |
| Condition | 0.167 | 0.07 | 0.37 | 7.57 | 19.26 |  |
| Condition + Condition * CS Type | 0.167 | 0.41 | 3.40 | 1.30 | 19.64 |  |
| Condition + Condition * Trial | 0.167 | 2.14e^-4^ | 0.001 | 2447.13 | 19.30 |  |
| Condition + Condition * CS Type + Condition  *  Trial | 0.167 | 0.001 | 0.01 | 422.16 | 19.28 |  |
| Condition + Condition * CS Type + Condition * Trial + Condition * CS Type * Trial | 0.167 | 1.19e^-6^ | 5.95e ^-6^ | 4.41e^5^ | 19.53 |  |
|  |  |  |  |  |  |  |
| Fear Potentiated Startle Responses |  |  |  |  |  |  |
| Null model (incl. CS Type, Trial, CS Type * Trial, Subject) | 0.167 | 0.93 | 62.40 | 1.000 |  |  |
| Condition | 0.167 | 0.06 | 0.32 | 15.01 | 2.94 |  |
| Condition + Condition * CS Type | 0.167 | 0.01 | 0.06 | 76.32 | 2.83 |  |
| Condition + Condition * Trial | 0.167 | 3.17e^-4^ | 0.002 | 2.92e^4^ | 1.67 |  |
| Condition + Condition * CS Type + Condition  *  Trial | 0.167 | 6.50e^-5^ | 3.25e^-4^ | 1.42e^4^ | 2.95 |  |
| Condition + Condition * CS Type + Condition *Trial + Condition * CS Type * Trial | 0.167 | 1.92e^-7^ | 9.60e^-7^ | 4.82e^6^ | 2.86 |  |
|  |  |  |  |  |  |  |
| Skin Conductance Responses |  |  |  |  |  |  |
| Null model (incl. CS Type, Trial, CS Type * Trial, Subject) | 0.167 | 0.83 | 24.00 | 1.00 |  |  |
| Condition | 0.167 | 0.16 | 0.95 | 5.21 | 2.32 |  |
| Condition + Condition * CS Type | 0.167 | 0.01 | 0.06 | 65.13 | 4.55 |  |
| Condition + Condition * Trial | 0.167 | 7.02e^-4^ | 0.004 | 1179.59 | 2.21 |  |
| Condition + Condition * CS Type + Condition  *  Trial | 0.167 | 5.23e^-5^ | 2.62e^-4^ | 1.58e^4^ | 2.79 |  |
| Condition + Condition * CS Type + Condition *Trial + Condition * CS Type * Trial | 0.167 | 4.23e^-7^ | 2.11e^-6^ | 1.96e^6^ | 2.98 |  |
| *Note.* All models, including the Null model, contain the variables CS Type, Trial, CS Type * Trial, and include a random intercept for every participant.  P(M) indicates the prior mode probabilities – we used non-informative uniform priors, so all models had equal prior probabilities. The P(M\|data) column shows the posterior model probabilities. BF_M_ shows the change from prior to posterior model odds. The BF_01_ column lists the Bayes factor for the null model against each alternative model. Higher values in the BF_01_ column reflect a higher probability of the data given the null model compared to the alternative model. The error percentage lists the proportional error associated with the value of the Bayes Factor. | | | | | | |
